# UniProt: the Universal Protein Knowledgebase in 2023

**DOI:** 10.1093/nar/gkac1052

**Published:** 2022-11-21

**Authors:** The UniProt Consortium, Alex Bateman, Maria-Jesus Martin, Sandra Orchard, Michele Magrane, Shadab Ahmad, Emanuele Alpi, Emily H Bowler-Barnett, Ramona Britto, Hema Bye-A-Jee, Austra Cukura, Paul Denny, Tunca Dogan, ThankGod Ebenezer, Jun Fan, Penelope Garmiri, Leonardo Jose da Costa Gonzales, Emma Hatton-Ellis, Abdulrahman Hussein, Alexandr Ignatchenko, Giuseppe Insana, Rizwan Ishtiaq, Vishal Joshi, Dushyanth Jyothi, Swaathi Kandasaamy, Antonia Lock, Aurelien Luciani, Marija Lugaric, Jie Luo, Yvonne Lussi, Alistair MacDougall, Fabio Madeira, Mahdi Mahmoudy, Alok Mishra, Katie Moulang, Andrew Nightingale, Sangya Pundir, Guoying Qi, Shriya Raj, Pedro Raposo, Daniel L Rice, Rabie Saidi, Rafael Santos, Elena Speretta, James Stephenson, Prabhat Totoo, Edward Turner, Nidhi Tyagi, Preethi Vasudev, Kate Warner, Xavier Watkins, Rossana Zaru, Hermann Zellner, Alan J Bridge, Lucila Aimo, Ghislaine Argoud-Puy, Andrea H Auchincloss, Kristian B Axelsen, Parit Bansal, Delphine Baratin, Teresa M Batista Neto, Marie-Claude Blatter, Jerven T Bolleman, Emmanuel Boutet, Lionel Breuza, Blanca Cabrera Gil, Cristina Casals-Casas, Kamal Chikh Echioukh, Elisabeth Coudert, Beatrice Cuche, Edouard de Castro, Anne Estreicher, Maria L Famiglietti, Marc Feuermann, Elisabeth Gasteiger, Pascale Gaudet, Sebastien Gehant, Vivienne Gerritsen, Arnaud Gos, Nadine Gruaz, Chantal Hulo, Nevila Hyka-Nouspikel, Florence Jungo, Arnaud Kerhornou, Philippe Le Mercier, Damien Lieberherr, Patrick Masson, Anne Morgat, Venkatesh Muthukrishnan, Salvo Paesano, Ivo Pedruzzi, Sandrine Pilbout, Lucille Pourcel, Sylvain Poux, Monica Pozzato, Manuela Pruess, Nicole Redaschi, Catherine Rivoire, Christian J A Sigrist, Karin Sonesson, Shyamala Sundaram, Cathy H Wu, Cecilia N Arighi, Leslie Arminski, Chuming Chen, Yongxing Chen, Hongzhan Huang, Kati Laiho, Peter McGarvey, Darren A Natale, Karen Ross, C R Vinayaka, Qinghua Wang, Yuqi Wang, Jian Zhang

**Affiliations:** European Molecular Biology Laboratory, European Bioinformatics Institute (EMBL-EBI), Wellcome Genome Campus, Hinxton CB10 1SD, UK; Protein Information Resource, Georgetown University Medical Center, 2115 Wisconsin Ave NW, G1 level, Suite 040A, Washington, DC 20007, USA; Protein Information Resource, University of Delaware, Ammon-Pinizzotto Biopharmaceutical Innovation Building, Suite 147B, 590 Avenue 1743, Newark, DE 19713, USA; SIB Swiss Institute of Bioinformatics, Centre Medical Universitaire, 1 rue Michel Servet, CH-1211 Geneva 4, Switzerland

## Abstract

The aim of the UniProt Knowledgebase is to provide users with a comprehensive, high-quality and freely accessible set of protein sequences annotated with functional information. In this publication we describe enhancements made to our data processing pipeline and to our website to adapt to an ever-increasing information content. The number of sequences in UniProtKB has risen to over 227 million and we are working towards including a reference proteome for each taxonomic group. We continue to extract detailed annotations from the literature to update or create reviewed entries, while unreviewed entries are supplemented with annotations provided by automated systems using a variety of machine-learning techniques. In addition, the scientific community continues their contributions of publications and annotations to UniProt entries of their interest. Finally, we describe our new website (https://www.uniprot.org/), designed to enhance our users’ experience and make our data easily accessible to the research community. This interface includes access to AlphaFold structures for more than 85% of all entries as well as improved visualisations for subcellular localisation of proteins.

## INTRODUCTION

The UniProt databases enable the research community to explore the diversity of life as described by the complement of proteins expressed by each organism. The UniProt Knowledgebase (UniProtKB) comprises of the reviewed protein set (UniProtKB/Swiss-Prot), where each protein entry is linked to a summary of the experimentally verified, or computationally predicted, functional information added by our expert biocuration team, and the unreviewed UniProtKB/TrEMBL), in which entries are computationally annotated by automated systems. The UniRef databases cluster sequence sets at various levels of sequence identity and the UniProt Archive (UniParc) delivers a complete set of known unique sequences, including historical obsolete sequences. Data from selected resources are additionally integrated into UniProtKB records to add biological knowledge and associated metadata enabling the database to act as a central hub from which users can link out to 183 other resources. Community functional annotation adds further value to the entry annotations. The integration of these data and the manual curation of protein features, such as functional domains and active sites, amino acid variants, ligand binding sites and post-translational modifications (PTMs) in the UniProt record, provide our users with mechanistic insights into how, for example, specific variants can lead to disease or resistance to a drug or to a pathogen. In 2022, structural predictions were added from AlphaFold, a machine-learning system developed by DeepMind that predicts a protein's 3-dimensional (3D) structure from its amino acid sequence (1). More than 214 million entries now have AlphaFold structures available to view.

## PROGRESS AND NEW DEVELOPMENTS

### Managing the Sequence Space

**Figure 1:**
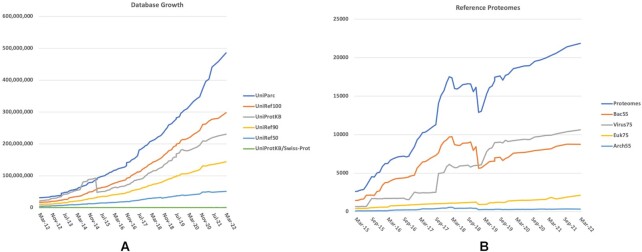
(**A**) Growth of UniProt databases over the last 10 years and (**B**) Growth of Reference Proteomes and taxonomic breakdown.

UniProt release 2022_03 contains over 227 million sequence records in UniProtKB. Figure 1A shows the continuing growth of all UniProt databases. The UniProtKB Proteomes portal (https://www.uniprot.org/proteomes/) provides access to more than 451 000 proteomes, which are sets of protein sequences originating from completely sequenced viral, bacterial, archaeal and eukaryotic genomes. Of these proteomes, 21 871 have been selected either by the research community or by computational clustering as reference proteomes, providing the complete, best annotated proteomes in their taxonomic group. Computationally selected reference proteomes are chosen based on a number of criteria, including the level of curation (reviewed versus unreviewed), protein name (e.g. names do not contain hypothetical or putative preferred), source organism (e.g. proteins from model organisms preferred) and length of protein. are stably maintained as the chosen representative unless a higher quality proteome is identified in that cluster. Figure 1B shows the growth of reference proteomes, the visible drop in numbers in 2019 being due to the initial implementation of redundancy removal procedure described below. The majority of these proteomes are derived from the translation of genome sequence submissions to the INSDC source databases (2)—ENA (3), GenBank (4) and the DDBJ (5), supplemented by genomes sequenced and/or annotated by groups such as Ensembl (6) and NCBI RefSeq (4). Viral proteomes are manually checked and verified and periodically added to the database.

The number of fully sequenced organisms continues to grow. Projects such as the Darwin Tree of Life (www.darwintreeoflife.org) and Earth BioGenome Project (www.earthbiogenome.org) are likely to greatly expand the coverage of the eukaryotic organisms, whilst metagenomic sequencing contributes to our coverage of prokaryotic organisms. We therefore need to continually evolve our strategy for the storage and annotation of this growing volume of data. The previously described algorithm (7) that detects redundant proteomes has been optimised to deal with a growing number of bacterial, archaeal and fungal species in UniProtKB. The algorithm creates clusters of proteomes at the species level, calculates proteome similarity within each cluster using pairwise alignment, calculates redundancy and uses a graph reduction method, until the graph only consists of non-redundant proteome nodes. In release 2022_03, there are 283 375 redundant proteomes (over 60% of all proteomes). These redundant proteomes continue to be available for searches and downloads in UniProt through UniParc.

### Expert curation

The summarizing of biological data obtained from the scientific literature remains critical for the production of UniProtKB. We continue to supply both human-readable free-text summaries and structured annotation appropriate for large-scale analyses and also increasingly provide training data sets for machine learning/artificial intelligence-based method development. Additionally, given that applied machine-learning methods are becoming ever more mature, we are actively exploring ways to integrate these approaches into the expert curation workflow and also to use some of these algorithms to automate annotation of experimentally uncharacterized proteomes.

### Small molecule ligands

We continued the standardisation of all small molecule annotations in UniProtKB using the chemical ontology ChEBI (8), focusing on biologically relevant (or ‘cognate’) ligands such as activators, inhibitors, and cofactors and their corresponding binding sites, knowledge of which is captured from the literature and protein structures in the Protein Data Bank (PDB/PDBe) (9,10). We structured and reannotated binding sites for cognate ligands using ChEBI, and we now use this reference vocabulary for all new ligand binding site annotations. At the time of writing (UniProt release 2022_03, August 2022), UniProt provides binding site annotations for 776 unique cognate ligands mapped to ChEBI for over 200 000 reviewed UniProtKB/Swiss-Prot protein sequence records and for over 17 million protein sequence records in UniProtKB/TrEMBL. This work makes cognate ligand data in UniProtKB (more) FAIR. It also allows powerful queries of ligand data taking advantage of the ChEBI chemical ontology hierarchy and chemical structure data, improves interoperability with other resources of cognate ligands (11–14), and provides better support for the development of computational approaches to predict protein-ligand interactions (15–17). This includes those being developed in the context of the UniProt metal binding challenge (see https://insideuniprot.blogspot.com/2022/02/the-uniprot-metal-binding-site-machine.html).

While restructuring ligand data, we also continued to improve knowledge of small molecule chemistry in UniProtKB through ongoing curation of enzyme and transporter functions using the Rhea knowledgebase of biochemical reactions (which uses ChEBI to represent reactants) (18,19). At the time of writing, UniProtKB includes annotations for 10 540 Rhea reactions, which are linked to 24 842 646 UniProtKB protein sequence records, including 226 101 reviewed protein sequence records of UniProtKB/Swiss-Prot. Our curation efforts around small molecule chemistry are guided by LitSuggest, an interactive platform to train and deploy advanced machine learning approaches for literature recommendation (20). UniProt curators have trained LitSuggest models using our own corpus of curated literature, which provides experimental evidence to link UniProtKB sequences and Rhea reactions. These models are able to recognize relevant literature with a very high degree of precision and provide a weekly digest for curators to assess and curate.

### Enhancing the human proteome

We continue to focus on improving both the sequence quality and annotation content of the human proteome, recurating sequences that are inconsistent, and creating records describing the products of newly identified protein-coding genes, such as micropeptides identified in what were thought to be long non-coding RNAs (21,22). New isoforms are added when verified by experimental data and a key focus is on the description of isoform-specific functional characterisation data. Conversely, sequences, including those of isoforms, have been removed when shown to be experimental artefacts or the result of erroneous gene model predictions. The GIFTS database (23) has been developed to provide a common framework for Ensembl and UniProt and enables both teams to read and comment on data, track entities between resources and support mappings between genes and the proteins which they encode. The coverage of this tool has now been extended to also enable improvements to the mouse, rat, zebrafish, maize and soybean proteomes.

At the time of writing, 93% of UniProtKB/Swiss-Prot canonical sequences are identical to their corresponding Ensembl protein sequences translated from the reference human genome and work is ongoing to understand the differences. For the remaining 7%, the discrepancies are due to a variety of reasons such as differing choice of initiator methionine or display of an alternative allele at a variant site. New data continue to emerge from methods such as ribosomal profiling which assists with identification of translation start sites, allowing many of these discrepancies to be resolved and improving consistency between the resources. Efforts are also being made to ensure consistency with the Matched Annotation from NCBI and EMBL-EBI (MANE) protein set (24). MANE is a collaboration between EMBL-EBI and NCBI to converge on human gene and transcript annotation and to jointly define a high-value set of transcripts and corresponding proteins. Investigation of sequences differing between UniProtKB/Swiss-Prot and MANE is underway, and these cases continue to be resolved or passed back to the MANE project where further investigation is needed. This work will ensure that a uniform set of protein sequences is available across multiple databases, aiding users as they navigate between different sequence resources.

### Community curation

**Figure 2.**
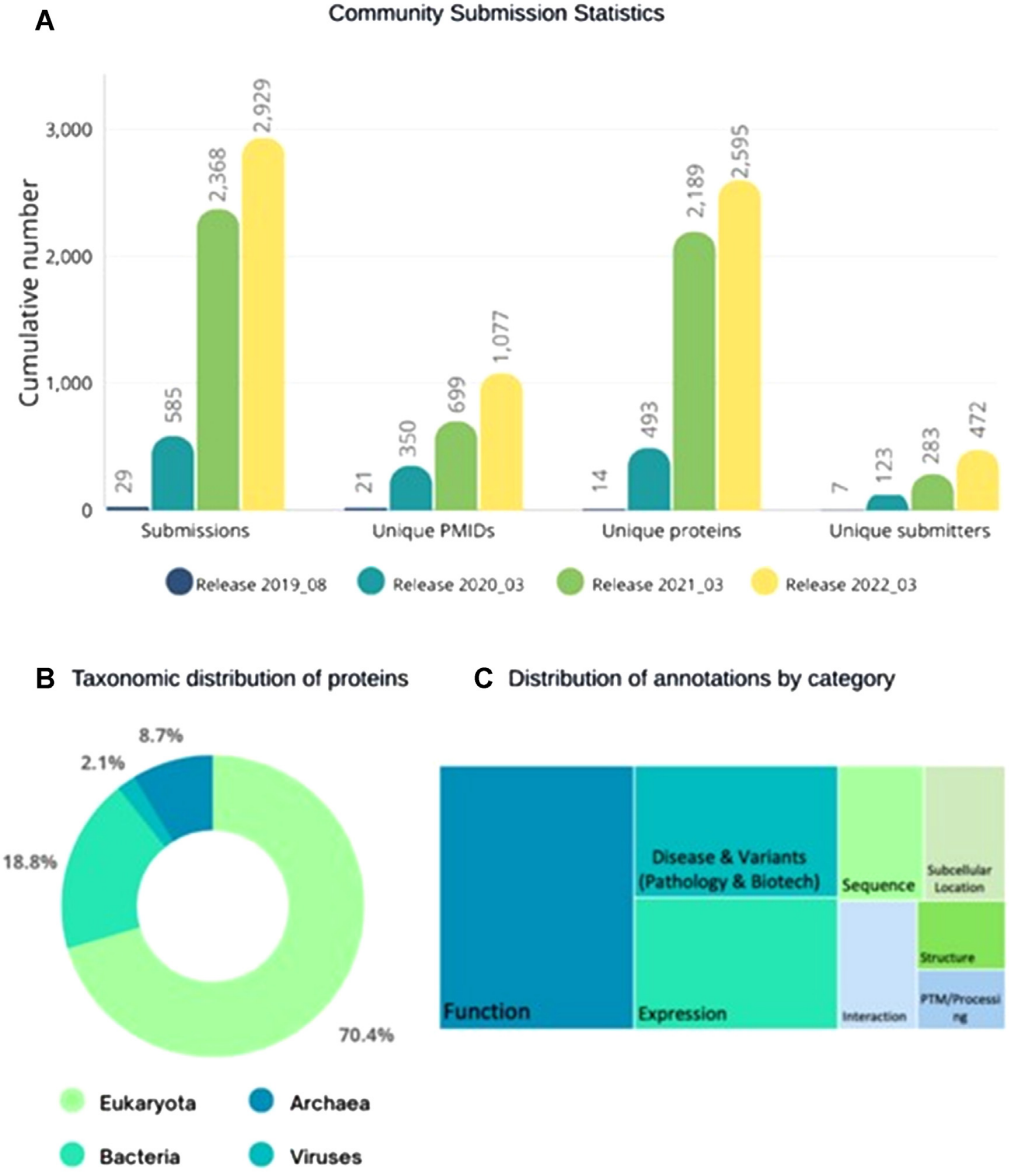
Statistics of UniProt crowdsourcing activity. (**A**) Cumulative number of submissions, unique publications and proteins covered, and number of contributors for selected releases. Release 2019_08 was the first release where community submissions appeared, and 2022_03 is the latest release at the time of this manuscript preparation. (**B**) Taxonomic distribution of unique protein entries that have at least one publication submitted by the community. (**C**) Block chart showing the relative distribution of annotations by categories.

The scientific community has been contributing publications and annotations to UniProtKB entries for the past three years. This crowdsourcing activity enables quick access to experimental information on unreviewed entries or those that could benefit from updates, independent of the database release cycle. Community submissions are quickly reviewed by a curator to ensure content is appropriate and that the publication has been linked to the relevant entry. Figure 2A shows a steady cumulative increase in submissions with respect to number of submissions, unique references, improved entries, and submitters. As of release 2022_03 (3 August 2022), there have been 2929 submissions from 472 unique users (https://community.uniprot.org/bbsub/STATS.html). The submissions add annotations to 2673 unique protein entries (44% reviewed and 56% unreviewed) from all super kingdoms (Figure 2B), including 1077 publications providing information on a variety of topics, but especially rich in publications related to Function (Figure 2C). The additional annotation provided by crowdsourcing has provided measurable benefit, for example by adding experimental information to proteins named ‘Uncharacterized protein’ (25). Those interested in contributing will find guidance on the home page for the project (https://community.uniprot.org/bbsub/home.html), which offers general information about ways to contribute (from within an entry or via batch submission), a link to the search page for community contributions (https://community.uniprot.org/bbsub/bbsubinfo.html), and citation of contributions. Contributors can cite their work using the link https://community.uniprot.org/bbsub/bbsubinfo.html?orcid = <ORCID>, where < ORCID > should be replaced by the contributor's ORCID. From within a UniProtKB entry, community curated publications can be accessed via the link called ‘community curation’, which appears on the top menu on the UniProtKB entry page. UniProt crowdsourcing is an invaluable source of relevant publications and annotations that helps to scale up curation. Accordingly, we plan to integrate community-added information directly into the protein entry view.

### Automatic annotation

As large-scale sequencing projects continue to add to the number of proteomes we describe in UniProt, our need to extend and refine our automated procedures for transferring information from experimentally characterized proteins in UniProtKB/Swiss-Prot to the unreviewed records in UniProtKB/TrEMBL increases. Information is added to unreviewed records by systems based on the protein classification resource InterPro (26), which categorizes sequences into protein families, and predicts the existence of functional domains and functionally relevant regions. The semi-automated rule-based computational annotation UniRule system (27) combines the detailed annotation found in the reviewed records in UniProtKB with the information on protein families predicted by InterPro, in order to create rules for propagating annotation to the unreviewed proteins in the database. The number of UniRules has now increased to 8280 (Release 2022_03) and, as part of each release of UniProtKB, every unreviewed UniProtKB/TrEMBL record is evaluated against every UniRule, and where the record meets the conditions of the rule, the associated annotations are added or updated as appropriate.

To complement the process of manually creating UniRules, we have, as previously described (28), developed the Association-Rule-Based Annotator (ARBA), a multiclass, self-training annotation system for automatic classification and annotation of UniProtKB proteins. ARBA generates human-readable rules for each release which are made available at https://www.uniprot.org/arba/. ARBA rules predict protein names, catalytic activities, EC numbers, cofactors, pathways, subcellular locations, subunits, functions, family membership and GO terms. For each annotation value ARBA aggregates the prediction models into one comprehensive rule. In release 2022_03 ARBA has generated 27 338 rules. In combination, UniRule and ARBA rules have annotated 121 008 011 protein records in UniProtKB/TrEMBL (53.4%) in release 2022_03.

We are also working to improve and standardize the nomenclature of protein names, particularly those which are only described as ‘uncharacterized’ or by an ORF or cDNA designator in the database. Using InterPro we can expand these names to at least include a description of the protein's properties, e.g. ‘SH3 domain-containing protein’. We are collaborating with a research team at Google, who developed a deep learning model that predicts a description for every protein in UniProtKB/TrEMBL. These descriptions have been evaluated by expert curators and feedback incorporated into subsequent rounds of model building. In a first step we are going to use to use these descriptions to annotate 55 million proteins (Release 2022_04) in UniProtKB/TrEMBL, that otherwise have no other description than ‘Uncharacterized protein’.

### Data integration

Over 85% of UniProt entries now contain a predicted protein structure, provided by AlphaFold, an artificial intelligence system developed by DeepMind that makes predictions of protein structures from their amino-acid sequences (1). An interactive molecular viewer displays the structure, coloured by the per-residue pLDDT confidence measure, which is estimated on a 0–100 scale, with higher scores corresponding to higher confidence. These data are updated with every release of the DeepMind dataset.

As previously described (28), UniProtKB continues to import and integrate large-scale datasets, mapping these data onto the appropriate protein sequence records, displaying the mappings via the ProtVista visualisation tool (29) and making them accessible via FTP and APIs. Updated datasets from clinically relevant sources of sequence variation (e.g. 100K genomes, gnomAD and ClinVar SNPs) are mapped to protein features and variants using a pre-calculated mapping of the genomic coordinates for the amino acids at the beginning and end of each exon and the conversion of UniProt sequence positional annotations to their genomic coordinates (30). Unique and non-unique peptides identified by mass spectrometry proteomic data deposited through the ProteomeXchange Consortium (31) are also mapped to the underlying protein sequence and can be taken as evidence that a protein has been validated (PE = 1) using a variation of the HPP guidelines (32). In brief, at least two unique peptides of seven amino acids or more or, for proteins where this cannot be achieved due to sequence constraints, one unique peptide of ten amino acids or more has been mapped to a protein. Work is now ongoing to extend this to the import of high quality post-translational modifications, initially limiting this to phosphorylation sites, which again will be mapped to the relevant sequence and visualised in the ProtVista viewer. Details of the effect of amino acid mutations on protein interactions curated by members of the IMEx Consortium, of which UniProt is an active member, have also been imported and can be visualized via the ProtVista viewer (33,34). All data is accessible and downloadable using our new programmatic access interface (API) (see below).

### Website

With the increasing volume and complexity of our data, we have to make concomitant changes to the way in which we present information to our different user communities and enhance and diversify our search capabilities. To that end, we have released the new UniProt website, which builds upon the strengths of our previous design, with an emphasis on a responsive design, improved search and navigation, new tool interfaces and a new programmatic access interface (API). The website adopts a modular approach and separates the front-end (Web user interface) from the back-end (API). This improves responsiveness while remaining scalable and eases maintenance. A new caching logic has been implemented that supports simple and complex popular queries. The new API, which provides programmatic access, is fully documented with examples in various popular programming languages and is available from https://www.uniprot.org/help/api.

### Responsive design

The new responsive design provides better support for accessing UniProt data. As UniProt is used on a variety of devices, it is essential that our layout adapts to different device screen sizes. The size of the elements on the page adjusts itself depending on the available space on each user's device in order to present our data in an adapted way according to responsive design principles. Full data visualisations are rendered in the entry view on bigger screens, whereas on smaller screens, where device resources tend to be more limited, we reduce these visualisations in favour of only presenting the raw tables of data (Supplementary Figure 1).

### Improved search and navigation

A powerful search engine allows searching for any piece of information (e.g. a gene name) stored in UniProt and/or any combination of terms (e.g protein name and organism name / ‘Apolipoprotein E human’). Users can search and perform complex queries in the ‘Advanced search’ functionality which contains a range of fields for more detailed search. The results pages now include more filters to help users narrow down the results set and tailor it to their use case. The results table can be customised by adding columns with additional data and it can also be downloaded in various formats. The ‘Share’ and ‘Download’ functionalities allow for URLs and APIs to be built to help users retrieve data in a number of different formats (text, xml, etc.), as well as the newly added JSON format.

**Figure 3:**
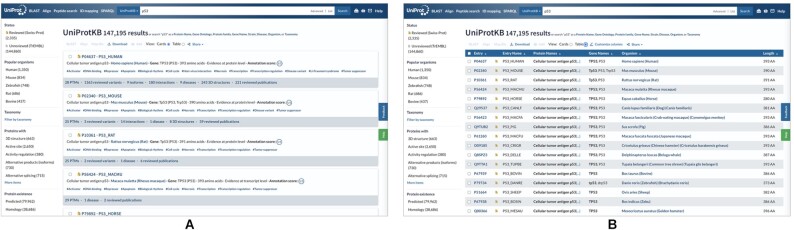
(**A**) Card view of results following a search of the UniProt website and (**B**). Table view of results following the same search.

The redesign took into consideration feedback from different user groups to improve the experience of navigating search results. This is reflected in the option to view results in the card view (Figure 3A), in addition to the existing table view (Figure 3B). The card view provides a quick overview of the annotations available for entries in the results (e.g. available information on variations, structures, and post-translational modifications). This view is also now used when users search for specific information on one or a group of proteins, for example for proteins referenced in a specific citation or those assigned to a specific subcellular location, as it provides immediate access to the supporting data. We have, in response to user feedback, re-ordered the presentation of information in the single entry view, grouping information by theme and with the relevant data visualisation view alongside.

### New tools dashboard

As part of the redesign, the Tools dashboard was created to improve workflows and allow for new ways of using and revisiting data. The Tools dashboard displays a user's list of tool jobs, both those currently running and previously completed queries. This allows users to submit multiple jobs simultaneously and to easily navigate between them. The user can change the name of their job to make it easier to identify, and they can also save, resubmit and delete jobs. All of this has been made persistent, so that earlier jobs are automatically stored for a defined period of time.

For BLAST (4) and Align tools, we offer several improvements. We now provide additional filters for BLAST results, such as filtering based on protein existence and sequence length. Moreover, users can now view score distribution of their BLAST results set, and they can also acquire the script for running the same job programmatically. The Align tool enables users to make multiple sequence alignments which can now be viewed in two ways—the Wrapped view allows for a quick scan of the alignment, and the Overview allows researchers to zoom in/out and move through the sequences in a user-defined manner. In either view, the user can select annotations for one of the proteins in the alignment and display these sequence- or structurally-defined features on the sequence alignment. The alignment visualisation is the same as that used to view BLAST pairwise alignments, thus improving the consistency in the visualisations throughout the website. With the same reasoning, the viewers used to overlay interactions and sequence features in this visualisation are the same as the feature viewers throughout UniProtKB entry pages. Additionally, the percent identity match of the aligned sequences can now also be viewed via a new tab on the Align results page.

### Interactive visualisations in entry pages

Multiple visualisations are now available throughout the main entry page to enable the exploration of protein features, e.g binding sites or catalytic residues, in the context of the sequence of the protein. The features presented in each visualisation are appropriate for the section of the entry page they are integrated into, allowing for a cleaner view of the features grouped by category. We also integrate a structure visualisation in the entry page that allows the user to view 3D-structures from PDB, as well as structure predictions from AlphaFold integrated with all other data in the entry page. Additionally, the user can also see the full ProtVista feature viewer in a separate tab which integrates all visualisations with the structure viewer, allowing a unified view of protein sequence features.

### SwissBioPics

**Figure 4.**
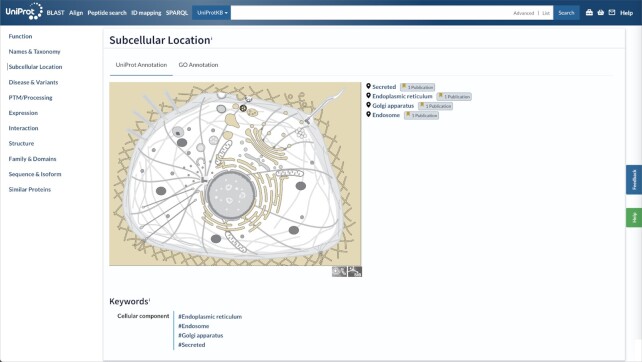
UniProtKB entry CL18A_HUMAN (UniProtKB: A5D8T8) shows an embedded SwissBioPics image: the generic animal cell (Eumetazoa) is selected based on organism taxonomy. It contains 71 interactive locations, of which the endoplasmic reticulum, Golgi apparatus, endosome and secretory space are highlighted using annotations from the UniProt entry.

Additional visualisations of the subcellular localizations of proteins are now provided in UniProtKB using the web component of SwissBioPics (www.swissbiopics.org) (35), a library of interactive cell images in which subcellular locations and organelles are mapped to terms from the UniProtKB controlled vocabulary (www.uniprot.org/locations/) and the ‘Cellular Component’ branch of the Gene Ontology (Figure 4). SwissBioPics describes a broad range of cell types from all branches of the tree of life—at the time of writing it provides images for 325 250 of 568 002 UniProtKB/Swiss-Prot records as well as many millions of UniProtKB/TrEMBL entries—and visualisations for 342 of the 561 terms from the UniProtKB subcellular location controlled vocabulary.

## CONCLUSION

As the volume of whole genome sequencing data available to the research community keeps on increasing, UniProt has continued to react and ensure we offer our users quality protein sequence data which has been annotated to the highest possible standard. We are adapting our data input pipeline to ensure that we present a reference proteome for each taxonomic grouping to the research community. We continue to evaluate the scientific literature and expertly curate individual records with relevant experimental data and use that data as a training cohort to enable the annotation of proteins which have not yet been biochemically characterized. We are working to further develop methods to enable this transfer of annotation and are actively collaborating with the machine-learning community to further enhance the information available on millions of uncharacterised proteins.

UniProtKB continues to act as a central hub of information with data from many external resources, including community annotation contributions, being imported, integrated and displayed alongside that added by the UniProt team. We have, in this period, produced and released a new website to facilitate the search and retrieval of these increasingly rich datasets with new and improved graphical visualisations to enhance the user experience. Upgraded APIs improve computational access to this information.

We greatly value the feedback and annotation updates from our user community. Please send your feedback and suggestions via the contact link on the UniProt website (https://www.uniprot.org/contact).

## DATA AVAILABILITY

UniProt releases are published every eight weeks. We provide customizable views and downloads in a range of formats via the website, and file sets at the FTP site (www.uniprot.org/downloads), and supply users with a number of different options for computational access to the data (www.uniprot.org/help/programmatic_access). These include the website RESTful Application Programming Interface (API), stable URLs that can be bookmarked, linked, and reused, the SPARQL API that allows users to perform complex queries across all UniProt data and also other resources that provide a SPARQL endpoint and a Java API.

## Supplementary Material

gkac1052_Supplemental_File
