# Supplementary figures and images for: UniProt: the Universal Protein Knowledgebase in 2023
Source: Nucleic Acids Res. 2022 Nov 21;51(D1):D523–31. doi: 10.1093/nar/gkac1052 (PMC9825514; doi:10.1093/nar/gkac1052)

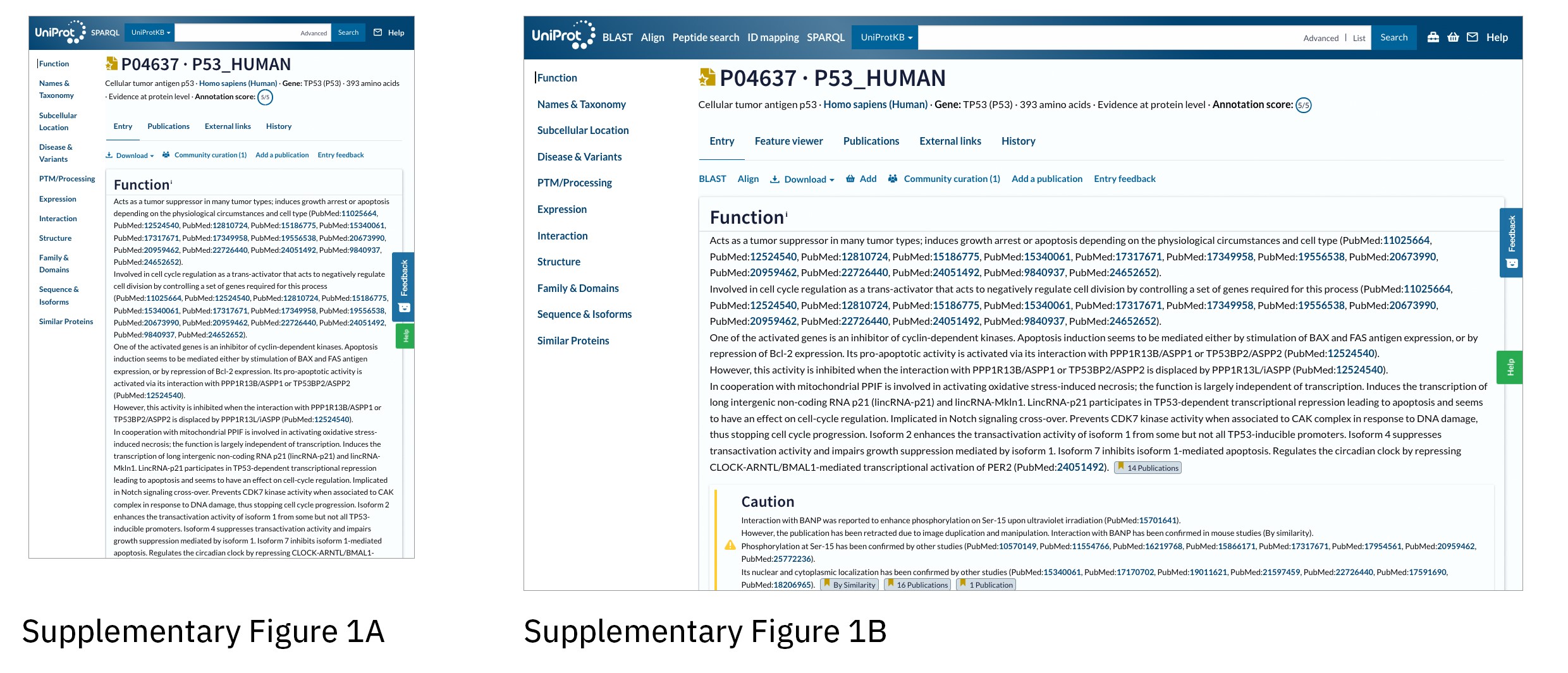

Supplement: gkac1052_Supplemental_File [file gkac1052_supplemental_file.jpeg]
